# Tryptophan, glutamine, leucine, and micronutrient supplementation improves environmental enteropathy in Zambian adults: a randomized controlled trial

**DOI:** 10.1093/ajcn/nqz189

**Published:** 2019-08-28

**Authors:** John Louis-Auguste, Ellen Besa, Kanekwa Zyambo, Derick Munkombwe, Rosemary Banda, Themba Banda, Alastair Watson, Jordi Mayneris-Perxachs, Jonathan Swann, Paul Kelly

**Affiliations:** 1 Blizard Institute, Barts and The London School of Medicine and Dentistry, Queen Mary University of London, London, United Kingdom; 2 Tropical Gastroenterology & Nutrition Group, University of Zambia School of Medicine, Lusaka, Zambia; 3 Department of Pharmacy, School of Health Sciences, University of Zambia, Lusaka, Zambia; 4 Norwich Medical School, Norwich, United Kingdom; 5 Department of Diabetes, Endocrinology, and Nutrition, Girona Biomedical Research Institute, Dr Josep Trueta University Hospital, Centre for Physiopathology of Obesity and Nutrition (CIBEROBN), Girona, Spain; 6 Department of Medical Sciences, Faculty of Medicine, University of Girona, Girona, Spain; 7 Division of Integrative Systems Medicine and Digestive Diseases, Faculty of Medicine, Imperial College London, London, United Kingdom

**Keywords:** environmental enteropathy, amino acids, micronutrients, MTOR, confocal endomicroscopy, morphometry, clinical trial, metabolomics, Zambia

## Abstract

**Background:**

Environmental enteropathy (EE) refers to villus blunting, reduced absorption, and microbial translocation in children and adults in tropical or deprived residential areas. In previous work we observed an effect of micronutrients on villus height (VH).

**Objective:**

We aimed to determine, in a randomized controlled trial, if amino acid (AA) or multiple micronutrient (MM) supplementation can improve intestinal structure or barrier dysfunction in Zambian adults with EE.

**Methods:**

AA (tryptophan, leucine, and glutamine) and/or MM supplements were given for 16 wk in a 2 × 2 factorial comparison against placebo. Primary outcomes were changes in VH, in vivo small intestinal barrier dysfunction assessed by confocal laser endomicroscopy (CLE), and mechanistic (or mammalian) target of rapamycin complex 1 (MTORC1) nutrient responsiveness in lamina propria CD4^+^ lymphocytes.

**Results:**

Over 16 wk AA, but not MM, supplementation increased VH by 16% (34.5 μm) compared with placebo (*P* = 0.04). Fluorescein leak, measured by CLE, improved only in those allocated to both AA and MM supplementation. No effect was seen on MTORC1 activation, but posttreatment MTORC1 and VH were correlated (ρ = 0.51; *P* = 0.001), and change in MTORC1 was correlated with change in VH in the placebo group (ρ = 0.63; *P* = 0.03). In secondary analyses no effect was observed on biomarkers of microbial translocation. Metabolomic analyses suggest alterations in a number of microbial- and host-derived metabolites including the leucine metabolite β-hydroxy-β-methylbutyrate, which was increased by AA supplementation and correlated with VH.

**Conclusions:**

In this phase 2 trial, AA supplementation protected against a decline in VH over the supplementation period, and improved barrier function when combined with micronutrients. Leucine and MTORC1 metabolism may be involved in the mechanism of effect. This trial was registered at www.pactr.org as PACTR201505001104412.

## Introduction

Environmental enteropathy (EE) was first recognized as “tropical enteropathy,” a phenomenon of uncertain significance (1–3). There is now evidence that it underlies the growth impairment of millions of young people globally (4, 5), impairs the neurocognitive development of children (6), contributes to micronutrient deficiencies through impaired absorption of nutrients (7), and may blunt responses to oral vaccines (5, 8).

The definitive assessment of EE requires small intestinal biopsy (1, 9). Recent work has established that assessment of fecal inflammatory and permeability markers can be used to evaluate the severity of EE (10–12), and these markers correlate with ponderal growth (12, 13). Recent metabolomic studies have found that EE is associated with perturbations in choline and tryptophan metabolism (14, 15). Transcriptomic analysis of stool RNA has shown that transcripts related to cell adhesion and to responses to viral and other pathogens characterize EE (16). We have recently reported that confocal endomicroscopy can be used to quantify leakiness of the epithelium in EE (17). T cell–mediated inflammation is a feature of EE, but the drivers of the mucosal immune response are as yet unknown.

There are currently no established therapies for EE, and even preventive strategies have been unsuccessful to date. Water and sanitation trials have so far proved disappointing (18, 19). Antibiosis using rifaximin did not alter lactulose permeation in Malawian children (20). There is divergent evidence on the effects of antihelminthics (21, 22). Probiotics were ineffective (23), as was anti-inflammatory therapy using mesalazine (24). New treatments are needed (25). We previously reported preliminary evidence that micronutrient supplementation could improve villus morphology (26). There are data which suggest that glutamine can reduce intestinal permeability (27, 28), but there are also negative trial data (29). In view of this preliminary evidence, and evidence that tryptophan metabolism is perturbed in EE (14, 30, 31), we hypothesized that micronutrient supplementation and/or supplementation with glutamine, tryptophan, and leucine can ameliorate the mucosal pathology of EE through modification of immune cell nutrient-sensing systems such as mechanistic (or mammalian) target of rapamycin complex 1 (MTORC1). Here we report the results of that randomized controlled trial.

## Methods

### Participants and recruitment

Participants were recruited from Misisi, Lusaka, where we have previously conducted studies of EE (9, 17). Recruitment followed a 3-stage process, beginning with door-to-door invitations, followed by a series of focus group discussions, and then individual interviews before written informed consent. At the initial assessment, inclusion and exclusion criteria were assessed, a medical and social history questionnaire was administered, and a physical examination was carried out. A stool sample was provided to exclude helminths and protozoal infection. An HIV test (Determine, Abbott Diagnostics) was administered, with a confirmatory test (Unigold, Trinity Biotech) administered in positive cases. On the day of endoscopy, a blood sample was collected, and body composition assessment was performed using impedance and plethysmography (see below).

### Inclusion and exclusion criteria

Participants were eligible for recruitment if between 18 and 60 y of age; male or female; resident in B section, Misisi compound, Lusaka; and able to give informed consent. Exclusion criteria were pregnancy (by self-report), breastfeeding (by self-report), BMI <18 kg/m^2^, any antibiotic use within the previous 4 wk, regular non-steroidal anti-inflammatory drug use within the previous 4 wk, diarrhea within the previous 4 wk, significant comorbidity precluding endoscopy with sedation, therapeutic anticoagulation or bleeding diathesis precluding endoscopic biopsies, or lack of consent for HIV testing. Untreated helminth or protozoal infection, or helminth or protozoal infection treated within 6 mo, were also exclusion criteria; however, participants with nonpathogenic parasites were eligible (detailed in **Supplemental Table 1**).

### Trial design

The trial was designed as a parallel-group phase 2 trial in which multiple micronutrient (MM) or amino acid (AA) supplements were compared with respective matching placebos in a 2 × 2 factorial design. The study was powered for primary outcomes in HIV-negative individuals, although HIV-positive participants were also included so as to avoid discrimination in this close-knit community. We did not detect any effect of HIV status on any of the outcomes measured, and results are reported here combined, without reference to HIV status unless otherwise stated.

### Randomization

Each AA or matched placebo sachet and each MM or matched placebo pillbox was labeled with 1 of 4 letters (A, B, C, D for AA/placebo) or numbers (1, 2, 3, 4 for MM/placebo), respectively. Two numbers and 2 letters were randomly assigned by the manufacturer to the active supplements, with the other 2 representing the placebos. The supplements were then labeled accordingly, and the allocation code was supplied to the study statistician. The code was known to the manufacturer and to the study statistician and was only revealed to the investigators once the databases had been locked at the end of the trial.

After completing enrollment and baseline assessments, participants were randomly allocated in blocks of 16 by the study statistician to 1 of the 16 possible groups (4 possible AA allocations × 4 possible MM allocations) using the randomization algorithms available at www.randomization.com (accessed 9 October, 2015 by DM). Participants were therefore allocated to each of the 4 study arms in a 1:1:1:1 ratio.

### Interventions

The MM supplement (Immunace Original) and matched placebo were both manufactured by Vitabiotics Ltd. The AA supplement (29.9 g l-Gln, 2.79 g l-Leu, and 0.28 g l-Trp) and isocaloric taste-matched placebo were both manufactured by Glanbia Nutritionals Deutschland GmbH. Both were given daily for 16 wk (112 d). Participants were advised to take both supplements with their main meal every day, at any time of day, starting the day after the baseline endoscopy. The composition and presentation of the active supplements and matching placebos are given in **Tables 1** and **2**.

**FIGURE 3:**
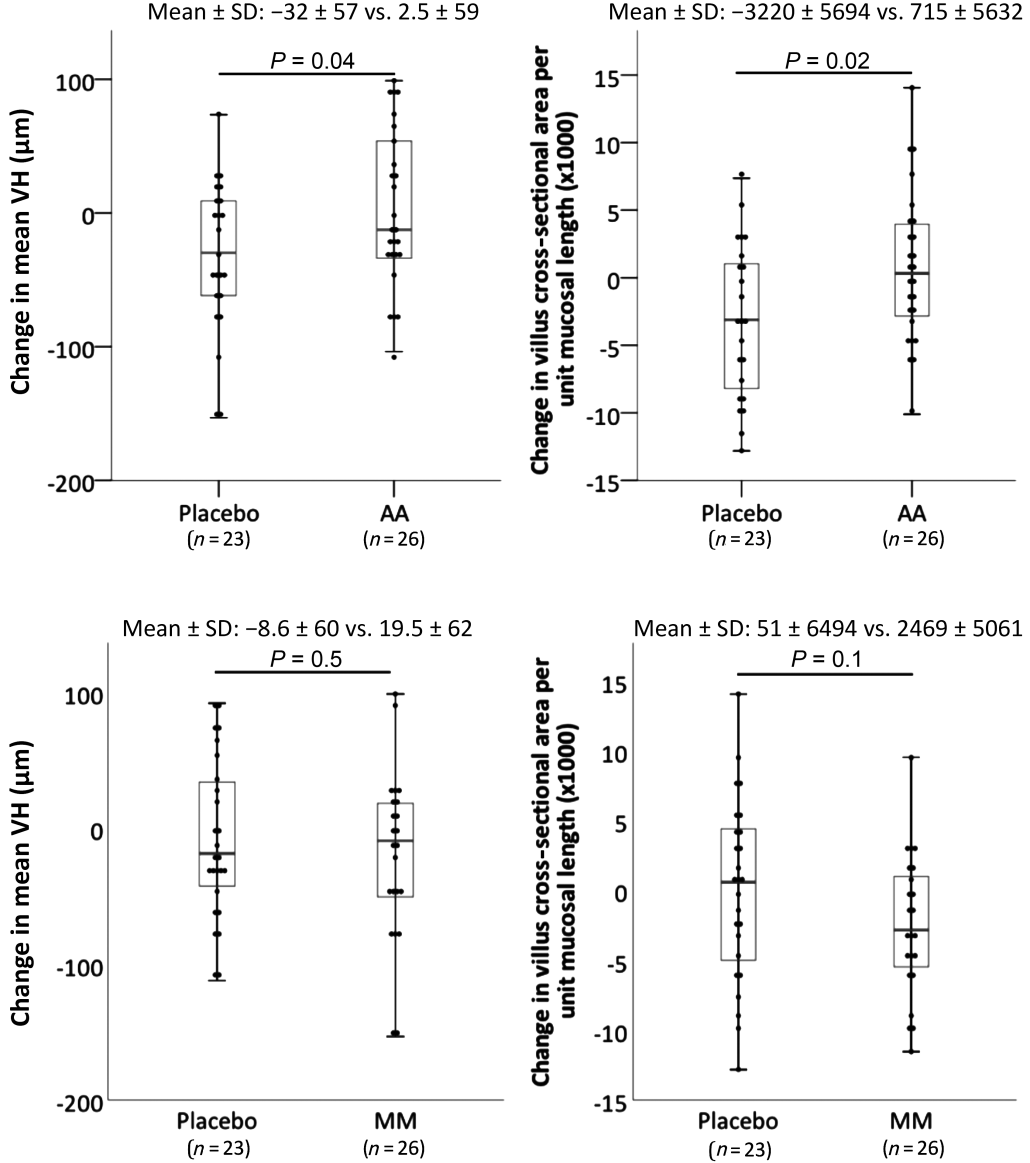
Change in VH and villus cross-sectional surface area per unit length of mucosa after AA (top panels) or MM (bottom panels) supplementation compared with placebo. Mean ± SD changes are given together with Tukey's hinges. Two-tailed independent-samples *t* test, with normality confirmed by the Shapiro–Wilk test. AA, amino acid; MM, multiple micronutrient; VH, villus height.

**FIGURE 4:**
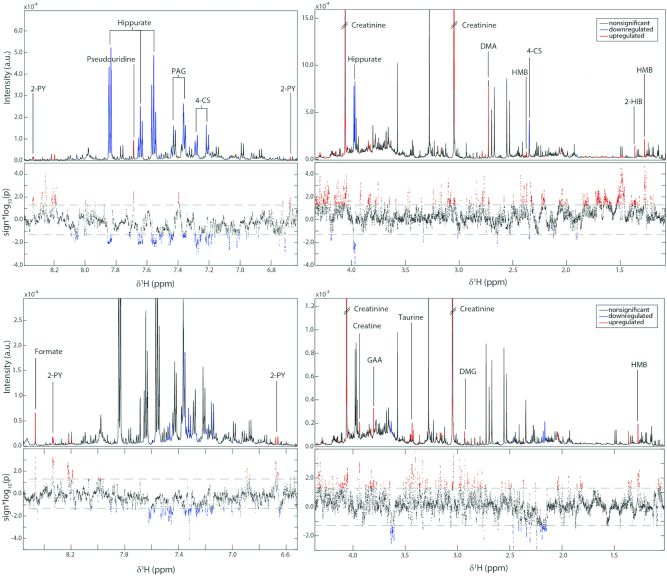
Associations between the urinary metabolic profile and MTORC1 (upper panels) or VH (lower panels) based on the OPLS models. The NMR spectra are divided into 2 (right and left) to show the full range of δ^1^H. For each comparison the upper section of the panel shows the average ^1^H NMR spectrum (intensity in arbitrary units) and the lower section shows a Manhattan plot which displays the −log10(*P* value) for each of the spectral variables. Statistically significant peaks from the OPLS models are colored red if positively associated with MTORC1 or VH, and blue if inversely associated. *n* = 46 for both VH and MTORC1 models. For details of statistical analysis see the Methods. 2-HIB, 2-hydroxyisobutyrate; 2-PY, *N*-methyl-2-pyridone-5-carboxamide; 4-CS, 4-cresyl sulfate; DMA, dimethylamine, DMG, dimethylglycine; GAA, guanidinoacetic acid; HMB, β-hydroxy-β-methylbutyrate; MTORC1, mechanistic (or mammalian) target of rapamycin complex 1; PAG, phenylacetylglutamine; OPLS, orthogonal projection to latent structures; VH, villus height.

**TABLE 1:** Composition of the micronutrient supplement^1^

	Average dose per tablet	Total daily dose	EC RDA	Multiples of EC RDA per daily dose
Vitamin A RE, IU/µg	2664/800	5328/1600	800	2
Vitamin D_3_, IU/µg	400/10	800/20	5	4
Vitamin E α-TE, mg	40	80	12	6.66
Vitamin K, μg	70	140	75	1.86
Vitamin C, mg	150	300	80	3.76
Thiamin, mg	18	36	1.1	32.72
Riboflavin, mg	6	12	1.4	8.58
Niacin NE, mg	27	54	16	3.38
Vitamin B-6, mg	10	20	1.4	14.28
Folic acid, μg	400	800	200	4
Vitamin B-12, μg	14	28	2.5	11.2
Pantothenic acid, mg	20	40	6	6.66
Iron, mg	8	16	14	1.14
Magnesium, mg	60	120	375	0.32
Zinc, mg	15	30	10	3
Iodine, μg	200	400	150	2.66
Copper, μg	500	1000	1000	1
Manganese, mg	4	8	2	4
Selenium, μg	180	360	55	6.54
Chromium, μg	100	200	40	5
l-Cystine, mg	40	80	N/A	N/A
l-Carnitine, mg	30	60	N/A	N/A
Citrus bioflavonoids, mg	30	60	N/A	N/A
β-Carotene (natural carotenoids), mg	3	6	N/A	N/A

^1^The total daily dose consisted of 2 tablets. The multiple micronutrient supplement included the following excipients: microcrystalline cellulose, hydroxypropylmethylcellulose, ethyl cellulose, propylene glycol, purified talc, titanium dioxide, iron oxides, glucose syrup, magnesium stearate, silicon dioxide, polyvinylpolypyrrolidone, acacia, sucrose, starch, tricalcium phosphate, dicalcium phosphate, medium-chain triglycerides, colloidal silica, maltodextrin, and butylated hydroxyanisole. The micronutrient placebo contained only these excipients. EC RDA, European Community RDA; IU, international units; N/A, not available; NE, niacin equivalents; RE, retinol equivalents; α-TE, α-tocopherol equivalents.

**TABLE 2:** Composition of the amino acid supplement^1^

	Active	Placebo
l-Gln	29.9 g	—
l-Trp	0.28 g	—
l-Leu	2.79 g	—
Maltodextrin	—	32.9 g
Sodium hydrogen sulfite (E222)	—	0.07 g
Total daily dose	33 g	33 g

^1^Sodium hydrogen sulfite was included as a bitterant in the placebo to approximate the slight bitterness of tryptophan.

### Primary endpoints

Three primary endpoints were evaluated in this phase 2 trial: *1*) change in villus height (VH) measured morphometrically in distal duodenal biopsies collected during endoscopy under sedation; *2*) change in in vivo small intestinal barrier leak by measuring fluorescein leak via confocal laser endomicroscopy (CLE); and *3*) change in distal duodenal lamina propria mucosal CD4^+^ T cell MTORC1 activity, measured by flow cytometry.

#### Morphometry

Endoscopic biopsies from distal duodenum were orientated under a dissecting microscope at the time of endoscopy before being fixed in formalin. Hematoxylin and eosin stained sections of paraffin-embedded tissue were then digitally imaged using a Hamamatsu digital slide scanner (Hamamatsu Photonics UK Ltd.). After identification of the villus–crypt boundary and mucosal length by the user, several parameters were measured: VH, villus width, crypt depth, villus perimeter per unit length of mucosa (as a measure of 3-dimensional villus surface area), and villus cross-sectional area per unit length of mucosa (as a measure of 3-dimensional villus compartment volume) (17).

#### CLE

**FIGURE 1:**
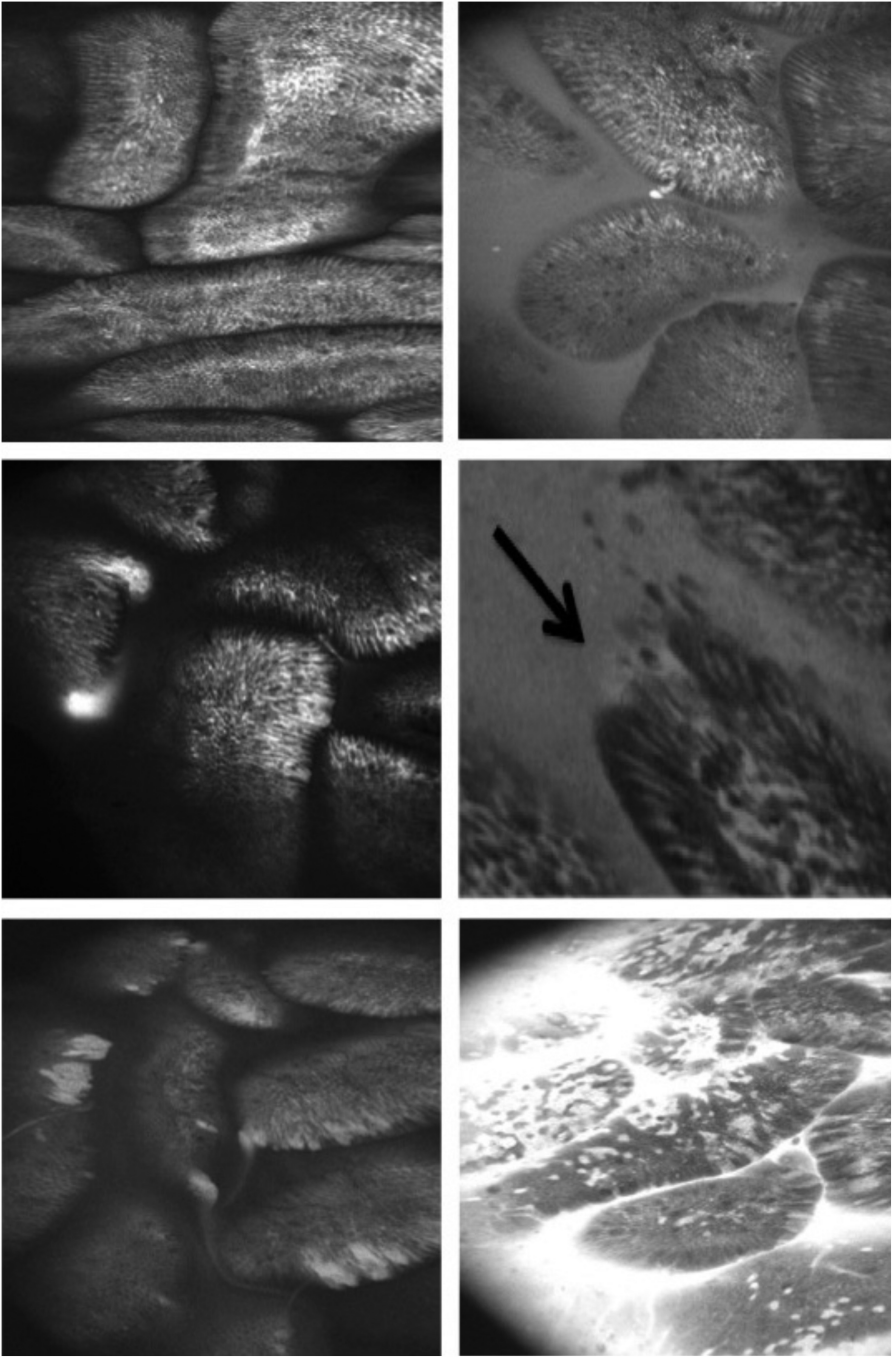
Examples of reproducible endomicroscopic signs of barrier dysfunction. Top left: intact mucosa; no leak; intervillous space is black (normal; Watson grade I). Top right: background luminal fluorescein (functional defect; Watson grade II) with a single apoptotic cell shedding event (nonpathological). Centre left: fluorescein plume with no mucosal defects (functional defect; Watson grade II). Center right: apical microerosion (multiple cell defect) with background fluorescein leakage (structural defect; Watson grade III). Bottom left: multiple microerosions with multiple fluorescein plumes (structural defect; Watson grade III). Bottom right: florid fluorescein leak within and out of the mucosa with multiple microerosions (structural defect; Watson grade III).

The small intestinal barrier was imaged in vivo using fluorescein-based CLE (Pentax) as previously described (17). The proportion of fields demonstrating fluorescein leak into the lumen (**Figure 1**) was scored by 2 independent blinded observers, whose reports demonstrated high interobserver agreement (ρ = 0.94; *P* < 0.001; **Supplemental Figure 1**).

#### Assessment of lamina propria CD4^+^ T lymphocyte MTORC1 activity

The ability of lamina propria CD4^+^ lymphocytes to respond to environmental nutrients through MTORC1 activity was assessed by measuring levels of its phosphorylated downstream transcription factor target, phosphor-4E-binding protein 1 (p4E-BP1) (31), via flow cytometry (see the **Supplemental Methods**). The percentage of CD4^+^ T cells responding to nutrient stimulation was calculated in FlowJo (TreeStar), using the “starved” cells as the control population.

### Secondary endpoints

Secondary endpoints measured included change in *1*) plasma markers of microbial translocation and inflammation; *2*) metabolomic profile; and *3*) anthropometry and body composition.

#### Plasma markers of microbial translocation and inflammation

Serum was stored at −80°C for analysis of LPS concentrations using the *Limulus* amoebocyte lysate assay (Associates of Cape Cod International Inc.) according to the manufacturer's instructions. C-reactive protein (CRP) and soluble CD14 (sCD14) (both from R&D Systems) concentrations were measured by ELISA according to the manufacturer's instructions. Serum glucagon-like peptide 2 (GLP-2; EMD Millipore Corporation) concentrations were measured by ELISA according to the manufacturer's instructions.

#### ^1^H NMR spectroscopy–based metabolic phenotyping

Urine samples were collected before endoscopy into a container with 1 mL 0.5% chlorhexidine, then stored at −80°C until transportation to London, United Kingdom on dry ice. Analytical details are given in the Supplemental Methods. Multivariate statistical approaches were used to interrogate the complex metabolic data set. This included principal component analysis to identify the main sources of variance in the data and to identify outliers. Orthogonal projection to latent structures (OPLS) discriminant analysis was performed to assess the metabolic variation between the different treatment groups (e.g., placebo compared with AA). Separate OPLS models were also constructed to illuminate metabolic variation associated with VH and MTORC1 activation. The predictive ability (Q^2^Y) of the models was calculated using a 7-fold cross-validation approach and their validity was assessed by permutation testing (1000 permutations; with a *P* value threshold of *P* < 0.05).

#### Nutritional assessments

Study participants had BMI, midupper arm circumference (MUAC), and grip strength measurements taken before and after the supplementation period. Body composition pre- and postsupplementation was assessed using whole-body air displacement plethysmography (BodPod, COSMED) and bioelectrical impedance (Tanita Corporation).

### Data analysis and statistical considerations

The study was powered to demonstrate the superiority of MM or AA supplementation over placebo in HIV-seronegative participants. We expected a 15% difference in morphometry to be associated with a similar degree of change in intestinal permeability, bacterial translocation, and metabolomic profile. Power and sample size calculations were based on morphometric data from 2 studies of adults with enteropathy in this population (17, 26). Sample size calculations were powered for main effects and assumed no negative interaction between AA and MM supplementation. To detect a difference of 15% between an intervention and the corresponding placebo would require 56 HIV-seronegative participants—28 participants given the supplement and 28 given the corresponding placebo (expected difference in VH = 30 µm; SD = 40 µm; power = 0.8; 2-sided α = 0.05). Therefore, 14 participants per combination of interventions was the minimum sample size. Allowing for a 20% dropout rate and 30% HIV-seropositive rate (17), we anticipated that 100 participants were required. Using the Shapiro–Wilk test, measures of morphometry and biomarkers were nonnormally distributed and are therefore reported as median [IQR]. However, measurements of change in morphometry, fluorescein leakage, and MTORC1 activation did not depart significantly from the normal distribution and are reported as mean ± SD.

### Clinical trial approvals

The study protocol and trial were approved by the University of Zambia Biomedical Research Ethics Committee (reference 007-11-14, dated 22 January, 2015), the Zambia Medicines Regulatory Authority (reference CT054/15), and the National Health Research Authority of Zambia (29 October, 2015), and registered with the Pan African Clinical Trials Registry (PACTR201505001104412). A favorable nonbinding opinion was also obtained from Queen Mary University of London Ethics of Research Committee (ref. QMERC2014/77).

## Results

**FIGURE 2:**
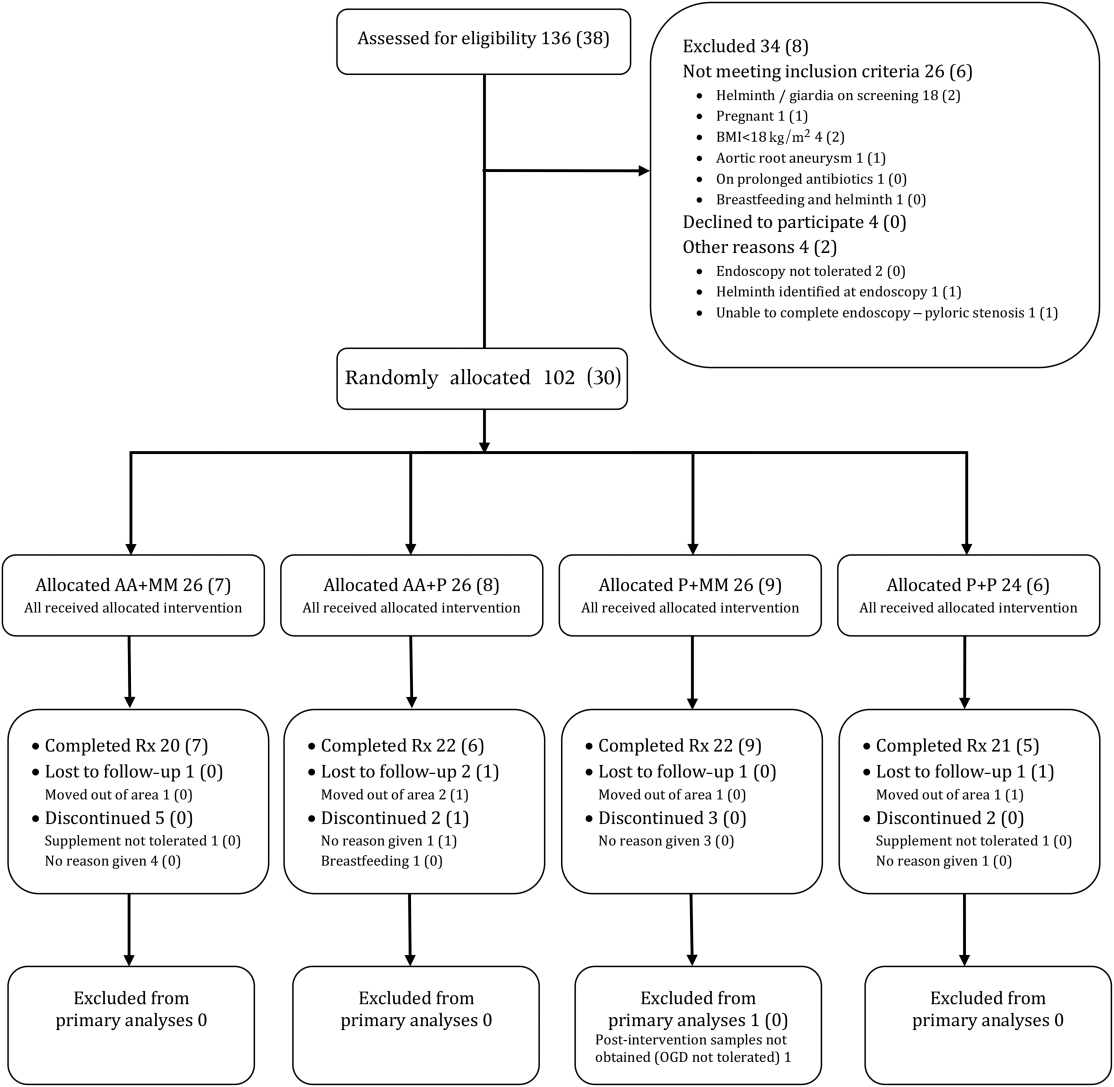
Consolidated Standards of Reporting Trials flow diagram of participants through the trial. Total numbers of participants are given, with the number of HIV-positive participants in parentheses. AA, amino acid; MM, multiple micronutrient; OGD, oesophagogastroduodenoscopy; P, placebo.

The trial was carried out between October 2015 and May 2016. The timing of recruitment was such that treatment was initiated in November–December 2015 (before the rainy season) and the final measurements after treatment were made in March–April 2016 (after the rainy season). One hundred and two participants were recruited and randomly allocated, and 85 were included in the final analysis (**Figure 2**). Compliance was high (**Supplemental Figure 2**).

### Participant characteristics

Age, sex, nutritional status (BMI, MUAC, fat distribution, grip strength), and other baseline measurements for primary endpoints were comparable between the treatment groups (**Table 3**).

**TABLE 3:** Baseline characteristics^1^

	AA arm		MM arm	
	AA (*n* = 42)	Placebo (*n* = 43)	MM (*n* = 42)	Placebo (*n* = 43)
Sex, M:F	16:26	11:32	12:30	15:28
Age, y	35 [28, 46]	42 [24, 51]	40 [21, 47]	40 [30, 46]
HIV seropositive	13 (31%)	14 (33%)	16 (38%)	11 (26%)
BMI, kg/m^2^	23.1 [19.9, 25.1]	22.3 [20.5, 27.7]	23.1 [20.2, 26.7]	22.0 [19.9, 25.9]
MUAC, cm	27.3 [25.4, 30.1]	28.5 [25.8, 32.0]	28.3 [25.4, 31.1]	27.9 [25.4, 31.3]
Grip strength, kg	31.8 [25.0, 39.2]	29.2 [27.1, 36.9]	30.7 [27.7, 36.1]	31.2 [25.7, 37.7]
Fat percentage, by impedance	24.1 [14.7, 30.0]	25.3 [17.6, 36.2]	25.2 [16.5, 31.6]	24.1 [15.7, 34.4]
Fat percentage, by plethysmography	24.5 [17.3, 34.3]	30.9 [19.3, 40.6]	30.3 [17.6, 37.6]	28.5 [17.3, 36.1]
VH, μm	185 [154, 217]	218 [201, 250]	202 [181, 252]	206 [170, 235]
CD, μm	163 [141, 196]	163 [150, 198]	157 [135, 194]	174 [152, 199]
VW, μm	230 [210, 263]	222 [197, 268]	221 [202, 253]	230 [205, 267]
VP, μm, per unit mucosa	865 [702, 1030]	987 [868, 1134]	931 [788, 1072]	913 [772, 1090]
VA, μm^2^, per unit mucosa	30,061 [23,036, 37,178]	32,328 [27,992, 40,093]	31,998 [23,619, 37,642]	31,288 [24,892, 37,200]
Fluorescein leak, % fields	85 [66, 91]	79 [57, 89]	80 [56, 91]	86 [75, 91]
p4E-BP1 positive LP CD4^+^, %	48 [41, 57]	60 [45, 66]	58 [46, 65]	49 [36, 62]
LPS, EU/mL	158 [106, 257]	155 [94, 232]	173 [108, 251]	150 [89, 244]
CRP, μmol/mL	1.9 [0.6, 3.6]	3.9 [1.1, 9.0]	2.2 [0.6, 7.2]	2.7 [0.9, 8.5]
sCD14, μmol/mL	1.8 [1.4, 2.4]	1.9 [1.4, 2.3]	1.7 [1.4, 2.4]	1.9 [1.5, 2.3]
GLP-2, ng/mL	0.76 [0, 1.4]	0.92 [0.5, 1.4]	0.75 [0.5, 1.5]	0.88 [0.5, 1.4]

^1^Values are given as median [IQR] unless otherwise stated. Fluorescein leak was defined as Watson grade II or III defects (see Methods and Figure 1 for further details), and scored as the percentage of unique endomicroscopic fields displaying leaks. The percentage of p4E-BP1 positive LP CD4^+^ represents the proportion of LP CD4^+^ that responds to nutrient stimulation in vitro (see the Methods for details). AA, amino acid; CD, crypt depth; CRP, C-reactive protein; F, female; GLP-2, glucagon-like peptide 2; LP CD4^+^, lamina propria CD4^+^ T cell; M, male; MM, multiple micronutrient; MUAC, midupper arm circumference; p4E-BP1, phospho-4E-binding protein 1 (marker of MTORC1 activation); sCD14, soluble CD14; VA, villus cross-sectional area per 100 μm muscularis mucosae; VH, villus height; VP, villus perimeter per 100 μm muscularis mucosae; VW, villus width.

### Primary endpoint: mucosal morphometry

Unfortunately, in several cases it was not possible for an experienced technician to obtain well-orientated sections suitable for morphometry, resulting in a reduction in the number of paired samples available for analysis. Nevertheless, there was a general reduction in villus and crypt measurements in all groups between the first and second biopsies, which may relate to seasonality (9). The reduction in VH and villus cross-sectional area was significantly less in participants given AA supplementation compared with placebo (**Table 4**; **Figure 3**). This effect of AA supplementation on VH was confirmed in linear regression models in which MUAC, fat mass, grip strength, and HIV status were included; only AA supplementation remained a significant contributor to the model (*P* = 0.03; **Supplemental Table 2**). MM supplementation had no effect on any morphometric measurement (Table 4). HIV status had no effect on morphometric parameters at baseline (data not shown) and had no effect on the change in VH over the supplementation period (mean ± SD: −28 ± 57 in HIV seropositives compared with −8.6 ± 61 in HIV seronegatives; *P* = 0.30).

**TABLE 4:** Changes in primary histological endpoints^1^

	AAs (Trp, Leu, Gln) vs. placebo			MMs vs. placebo		
Change in:	AA (*n* = 26)	Placebo (*n* = 23)	*P*	MM (*n* = 26)	Placebo (*n* = 23)	*P*
Villus height, μm	2.5 ± 59	−32.0 ± 57	0.04	−19.5 ± 62	−8.6 ± 60	0.53
Crypt depth, μm	−13.3 ± 39	−31.1 ± 45	0.15	−25.5 ± 43	−18.3 ± 43	0.56
Villus width, μm	−11.5 ± 60	−13.7 ± 138	0.94	−16.6 ± 72	−8.9 ± 126	0.80
Villus perimeter, μm, per 100 μm muscularis mucosae	−1.9 ± 180	−89.5 ± 159	0.08	−64.1 ± 137	−24.4 ± 203	0.43
Villus area, μm^2^, per 100 μm muscularis mucosae	715 ± 5632	−3220 ± 5694	0.02	−2469 ± 5061	50.8 ± 6494	0.14
Villus surface area:volume ratio	−0.0009 ± 0.0007	0.0003 ± 0.006	0.50	−0.00004 ± 0.006	−0.0006 ± 0.006	0.76

^1^Values are mean ± SD. Two-tailed independent-samples *t* test, with normality confirmed by the Shapiro–Wilk test. AA, amino acid; MM, multiple micronutrient.

### Primary endpoint: assessment of barrier dysfunction by CLE

Barrier leak was assessed using CLE (Figure 1). The median number of fields available for analysis was 72 at baseline and 98 after supplementation (IQR: 49–98 and 78–117.5, respectively). The mean ± SD change in the percentage of fields in which fluorescein leakage was detected was −10.1% ± 31% in the AA group compared with −5.1% ± 36% in the placebo group (*P* = 0.54). The mean ± SD change in the percentage of fields evaluated in which fluorescein leakage was detected was −11.3% ± 35% in the MM group compared with −4.3% ± 32% in the placebo group (*P* = 0.39). However, leak was reduced in the group allocated to AA + MM (*P* = 0.02 across all groups, *P* = 0.004 for interaction). The percentage of fields in which fluorescein leakage was observed was not correlated with morphometric or nutritional measurements (data not shown).

### Primary endpoint: assessment of nutrient sensing by lamina propria CD4^+^ T cells using p4E-BP1 as an index of MTORC1 activation

The ability of small intestinal lamina propria CD4^+^ T lymphocytes to respond to an environmental nutrient stimulus was assessed by flow cytometry. Both basal (starved cells) and peak (fed cells) MTORC1 activity [mean fluorescence intensity (MFI) of p4E-BP1^+^ CD4^+^ T lymphocytes after 90 min of nutrient deprivation or stimulation, respectively] declined over the course of the study. Basal mean ± SD MFI declined from 286.4 ± 84.7 presupplementation to 145.5 ± 55.7 postsupplementation (*P* < 0.001) but this did not differ by treatment allocation.

The mean ± SD proportion of CD4^+^ T lymphocytes that were nutrient responsive (i.e., p4E-BP1^+^ after nutrient stimulation) was 51.3% ± 16.3% presupplementation. The proportion of mucosal CD4 cells responding to the nutrient stimulus fell after the supplementation period in all groups. The mean ± SD change was −6.1% ± 16% in 9 participants allocated to AA supplementation, which did not significantly differ from −7.4% ± 21% in 16 participants allocated to placebo (*P* = 0.86). The mean ± SD change was −9.8% ± 19% in 16 participants allocated to MM supplementation, which did not significantly differ from −3.7% ± 20% in 14 participants allocated to placebo (*P* = 0.40). The small number of paired samples available for analysis reflects the harsh permeabilization required for p4E-BP1 staining, which as an undesired side effect resulted in the destruction of a variable proportion of lymphocytes.

Although MTORC1 activation was not affected by treatment allocation, it was strongly correlated with posttreatment VH (ρ = 0.51; *P* = 0.001; **Supplemental Figure 3**). The change in MTORC1 MFI over the period of supplementation was correlated with the change in VH in the placebo group (ρ = 0.63; *P* = 0.03), but not in the AA group (ρ = 0.26; *P* = 0.62). MTORC1 activation was not associated with sex, HIV status, fat-free mass, grip strength, or T cell activation as assessed by human leucocyte antigen-DR expression.

### Secondary endpoint: serum biomarkers

Serum concentrations of LPS, CRP, sCD14, and plasma GLP-2 did not differ between groups at baseline (Table 3). Changes in serum concentrations during the trial period did not differ in participants allocated to AA or placebo, or MM or placebo (**Supplemental Table 3**).

### Secondary endpoint: metabolomic analysis

Having observed the effect of AA supplementation on VH, the metabolic phenotypes were investigated for features that might explain this effect, or correlate with the changes observed. Significant OPLS models were obtained for the 3 active groups (MM, AA, and both) compared with placebo (**Tables 5** and **6**, **Figure 4**).

**TABLE 5:** Metabolomic changes in 3 active treatment groups compared with placebo^1^

	Increased with intervention	Reduced with intervention	*P* for model
MM (*n* = 15) vs. placebo (*n* = 18)	Pantothenate, HMB, PAG, 4-CS	NMNA, 3-HPHPA	0.01
AA (*n* = 16) vs. placebo (*n* = 18)	BAIBA, PAG, 4-CS, citrate	No significant changes	0.01
MM + AA (*n* = 17) vs. placebo (*n* = 18)	Pantothenate, succinate, HMB, PAG, fumarate, 2-PY	No significant changes	0.01

1 For details of statistical analysis see Methods. AA, amino acid; BAIBA, β-amino-isobutyric acid; HMB, β-hydroxy-β-methylbutyrate; MM, multiple micronutrient; NMNA, *N*-methylnicotinc acid; PAG, phenylacetylglutamine; 2-PY, *N*-methyl-2-pyridone-5-carboxamide; 3-HPHPA, 3-(3-hydroxyphenyl)-3-hydroxypropionic acid; 4-CS, 4-cresyl sulfate.

**TABLE 6:** Metabolomic changes over the supplementation period by treatment group^1^

	Increased over time	Reduced over time	*P* for model
MM pre vs. post (*n* = 18)	Pantothenate, HMB, succinate, 2-PY, NMND	BAIBA, 4-HH, 3-IS	0.01
AA pre vs. post (*n* = 16)	Citrate, myo-inositol, formate	Taurine, glycolate, *trans*-aconitate	0.01
MM + AA pre vs. post (*n* = 16)	Pantothenate, HMB, succinate, 2-PY, PAG, citrate, NMND, unknown	BAIBA, 2-HIB, choline, acetylcholine, taurine, hippurate	0.01
Placebo pre vs. post (*n* = 18)	No significant changes	No significant changes	N/A

^1^Significant metabolomic changes within treatment groups over time. For details of statistical analysis see Methods. AA, amino acid; BAIBA, β-amino-isobutyric acid; HMB, β-hydroxy-β-methylbutyrate; MM, multiple micronutrient; NMND, *N*-methylnicotinamide; PAG, phenylacetylglutamine; 2-HIB, 2-hydroxyisobutyrate; 2-PY, *N*-methyl-2-pyridone-5-carboxamide; 3-IS, 3-indoxyl sulfate; 4-HH, 4-hydroxyhippurate.

To explore drivers of our primary endpoints, the profiles were correlated with posttreatment VH and posttreatment MTORC1 activation. β-Hydroxy-β-methylbutyrate (HMB), dimethyglycine, creatine, creatinine, guanidinoacetic acid, taurine, *N*-methyl-2-pyridone-5-carboxamide (2-PY), and formate excretion were all positively correlated with VH. With respect to MTORC1 activation, HMB, 2-hydroxyisobutyrate, dimethylamine, pseudouridine, and 2-PY correlated positively, whereas 4-cresyl sulfate, hippurate, and phenylacetylglutamine (PAG) correlated negatively (**Table 7**). A summary of the origins and putative biological roles of these metabolites is given in **Supplemental Table 4**.

**TABLE 7:** Metabolomic changes correlated with postsupplementation VH and/or MTORC1 activity^1^

Outcome	Positively correlated	Negatively correlated	*P* for model
VH (*n* = 46)	HMB, DMG, creatine, creatinine, GAA, taurine, 2-PY, formate	No significant changes	0.01
MTORC1 (*n* = 46)	HMB, 2-HIB, 2-PY, pseudouridine, dimethylamine	4-CS, hippurate, PAG	0.01

1 Differentially excreted urinary metabolites correlated with posttreatment VH and posttreatment MTORC1 responsiveness. For details of statistical analysis see Methods. DMG, dimethylglycine; GAA, guanidinoacetic acid; HMB, β-hydroxy-β-methylbutyrate; MTORC1, mechanistic (or mammalian) target of rapamycin complex 1; PAG, phenylacetylglutamine; VH, villus height; 2-HIB, 2-hydroxyisobutyrate; 2-PY, *N*-methyl-2-pyridone-5-carboxamide; 4-CS, 4-cresyl sulfate.

### Secondary endpoint: nutritional assessment

Random allocation to MM supplementation had no impact on change in BMI, MUAC, fat-free mass (by impedance or plethysmography), or grip strength over this time period. There was also no difference in nutritional parameters by random allocation to AA supplementation (data not shown).

### Safety and adverse events

All supplements were well tolerated. The most frequent adverse events reported were feeling hungry, cough, diarrhea, nausea, vomiting, and dizziness. There were no serious adverse events and no deaths occurred. Adverse experiences were not more frequent in the AA or MM groups (**Supplemental Tables 5** and **6**).

## Discussion

EE is a subclinical alteration in small intestinal mucosal architecture and function, widely prevalent in the tropics and in disadvantaged communities. Previous trials have identified few interventions which consistently and successfully target any of the domains of pathophysiology, but nutritional or other interventions are needed. In this double-blinded randomized controlled trial, on a background of a seasonal reduction in VH and villus surface area, supplementation with AA but not MM increased VH and surface area compared with placebo. However, the effects on intestinal barrier function (assessed in vivo using CLE) were very modest and only seen in the AA + MM group. There was no effect on plasma biomarkers of microbial translocation. The pronounced correlation between posttreatment p4E-BP1 expression in lamina propria CD4^+^ cells and VH suggests that nutrient sensing by the MTORC1 pathway in the mucosal immune compartment plays a part in determining villus morphology, but it is also possible that improved absorptive capacity increases MTORC1 activation. The pathway of the effect of AA supplementation on mucosal architecture, if not through MTORC1, remains to be determined.

Our morphometric findings are in contrast to a secondary analysis of 1 of our previous studies which suggested that MM supplementation exerted a protective effect on small bowel histology (26). We cannot offer a robust explanation for this finding, but we speculate that seasonality may contribute to this discrepancy because the first study was conducted during the dry season and this study was conducted over the course of hot and rainy seasons. We and others have consistently demonstrated a seasonal variation in mucosal structure and/or intestinal permeability and microbial translocation (9, 26). A reduction in small bowel absorptive area over the course of the rainy season (which coincided with the supplementation period) was again observed. It is assumed that loss of absorptive capacity is related to the increased pathogen load observed during the rainy season, due to inadequate water and sanitation, but a reduction in dietary quality and quantity and increased food prices during this time may also be relevant, particularly with regards to nutrient-sensing pathways. With hindsight, it would have been better to have spread the trial over a full calendar year to include all seasons.

Confocal endomicroscopy is a novel tool for evaluating epithelial integrity in the intestinal mucosa (17, 32). We found it to be highly reproducible when used to assess leak severity measured by fluorescein efflux. There is evidence that fluorescein leak is a reflection of disturbed tight junction proteins and microerosions (17, 33). However, evaluation of details of epithelial integrity at a cellular level is time-consuming, and less reproducible than assessment of fluorescein leak. Although we found a modest response of epithelial leak to AA + MM, there was no response of a range of biomarkers of microbial translocation to the interventions. It is possible that mucosal barrier function and epithelial integrity are regulated by different factors to VH and villus surface area.

The lack of effect of HIV on mucosal morphometry and on barrier function may seem surprising. However, the data presented here are entirely consistent with our earlier work (9) which showed that only in advanced AIDS did VH fall below the range consistent with EE, whereas crypt depth was increased earlier in HIV infection. We believe that in industrialized countries HIV may affect villus morphology, but that in low- and middle-income countries any such subtle effect is undetectable alongside the EE, which is virtually ubiquitous.

Metabolomic analysis revealed a number of insights which may merit further investigation. A number of host- and gut microbial–derived metabolites were significantly correlated with VH and/or CD4^+^ T lymphocyte MTORC1 responses. For example, HMB was positively correlated with both MTORC1 and VH. HMB is a physiological leucine metabolite which has been demonstrated to enhance lean muscle mass and strength and decrease muscle protein breakdown via MTORC1 signaling (34). Interestingly, HMB increased with the MM supplement but not the leucine-containing AA supplement, suggesting that such changes were not driven by increased leucine intake. HMB has been previously observed to increase after zinc supplementation in children from Brazil (unpublished data). The gut microbial–host cometabolites 4-cresyl sulfate, PAG, and hippurate were all negatively associated with MTORC1. These metabolites have been found to be excreted in greater amounts by stunted children and are thought to arise from an overgrowth of bacteria and malabsorption in the small intestine preventing their precursors from being absorbed, thus increasing their bioavailability for microbial processing (35). Further work is required to assess their direct potential to activate MTORC1.

The nicotinamide/NAD^+^ pathway has been previously observed to be perturbed with chronic undernutrition and EE (14, 15). NAD^+^ is key for many metabolic processes including energy generation but is also important for DNA repair processes. NAD^+^ can be synthesized from the dietary precursors nicotinic acid (Preiss-Handler pathway), nicotinamide and nicotinamide riboside (salvage pathway), or via tryptophan (de novo biosynthesis pathway). Of note, either tryptophan or nicotinic acid was supplemented in 3 of the 4 trial arms. *N*-methylnicotinamide (NMND), a urinary marker of NAD^+^ abundance, has been positively associated with catch-up growth, whereas *N*-methylnicotinic acid (NMNA; trigonelline) excretion has been found to be reduced in wasted and underweight children. NMNA is a dietary constituent found in legumes and coffee. Although nicotinic acid is not methylated to NMNA in vivo, 1 study observed that when niacin-deficient rats received bound nicotinic acid (niacytin) from cereals, a prolonged excretion of NMNA followed. A defect in small intestinal permeability was implicated in the mechanism. In the present study, MM supplementation [containing nicotinic acid (niacin)] increased the excretion of NMND and its breakdown product 2-PY and decreased the excretion of NMNA. In addition, 2-PY was positively associated with VH and MTORC1 activation.

The tryptophan–indoleamine 2,3-dioxygenase (IDO)–kyneurine immunometabolic axis is currently a subject of great interest. Tryptophan is partly metabolized through host IDO 1 and 2 to produce kynurenines, and this pathway may be subverted by microbial tryptophan uptake and metabolism. High levels of IDO activity promote regulatory immune responses and T_reg_ differentiation and inhibit T_eff_ differentiation (36, 37). In stunted children with EE, plasma tryptophan concentrations are correlated with growth (38), and the kynurenine to tryptophan ratio (KTR, with high ratios indicative of high IDO activity) is correlated with systemic markers of inflammation and inversely correlated to growth (i.e., high KTR and presumed greater IDO activity are associated with greater stunting) (30), suggesting that microbial manipulation of tryptophan signaling and inhibition of kynurenine production may be relevant in the pathophysiology of EE. The metabolic differences observed in this study also suggest a possible role for tryptophan signaling in modulating the villus compartment and immune cell nutrient sensing.

A number of other metabolites correlated with VH, MTORC1 activity, or the interventions were identified. These can be broadly classed as AA–derived (e.g., taurine, 4-cresyl sulfate, PAG), tricarboxylic cycle intermediaries (e.g., succinate, formate), or microbial coproducts (e.g., pseudouridine, hippurate). Although some of these have been identified in undernourished/stunted populations previously, the significance of these findings in this study is unclear.

In recent reviews, it has been proposed that measurement of EE should be considered in several domains—villus morphology, permeability, microbial translocation, mucosal inflammation, systemic inflammation, malabsorption, and changes in the intestinal microbiota (35). Our data suggest that therapies for EE may have a preferential impact on 1 of these domains (villus morphology) without necessarily having a significant impact on the others. Indeed, our previous work has suggested that the observed villous atrophy may in fact have a protective role in EE (17). This suggests that VH, itself determined by enterocyte replication, migration, and apoptosis as well as stromal cells, might be rate-limited by the availability of nutrients such as glutamine, leucine, and tryptophan.

In summary, a safe and well-tolerated AA supplement in adults with EE resulted in increased VH (i.e., changes in gut mucosal structure), but these changes were not matched by improvements in intestinal barrier function, and intestinal barrier function only improved in the AA + MM group. There was consistent evidence of a correlation between VH and MTORC1 responsiveness in lymphocytes. Further work is required to determine whether modest improvements in small intestinal structure have relevant physiological benefits, such as improved absorption of nutrients. Given the promising effects of leucine in improving muscle mass in other contexts (39), work on AA/MM supplementation could provide benefits in >1 physiological system.

## Supplementary Material

nqz189_Supplemental_File

## Notes

Supported by CORE, UK (to JL-A) and Bill & Melinda Gates Foundation grant OPP1066118 (to PK). The micronutrient supplement and matching placebo were generously manufactured and donated by Vitabiotics Ltd, UK.

Supplemental Tables 1–6, Supplemental Figures 1–3, and Supplemental Methods are available from the “Supplementary data” link in the online posting of the article and from the same link in the online table of contents at https://academic.oup.com/ajcn/.

Data described in the article (an anonymized database) will be made available upon request to the corresponding author pending application and approval.

Abbreviations used: AA, amino acid; CLE, confocal laser endomicroscopy; CRP, C-reactive protein; EE, environmental enteropathy; GLP-2, glucagon-like peptide 2; HMB, β-hydroxy-β-methylbutyrate; IDO, indoleamine 2,3-dioxygenase; KTR, kynurenine to tryptophan ratio; MFI, mean fluorescence intensity; MM, multiple micronutrient; MTORC1, mechanistic (or mammalian) target of rapamycin complex 1; MUAC, midupper arm circumference; NMNA, *N*-methylnicotinic acid; NMND, *N*-methylnicotinamide; OPLS, orthogonal projection to latent structures; p4E-BP1, phospho-4E-binding protein 1; PAG, phenylacetylglutamine; sCD14, soluble CD14; VH, villus height; 2-PY, *N*-methyl-2-pyridone-5-carboxamide.
