# Supplementary material for: Tryptophan, glutamine, leucine, and micronutrient supplementation improves environmental enteropathy in Zambian adults: a randomized controlled trial
Source: Am J Clin Nutr. 2019 Aug 28;110(5):1240–52. doi: 10.1093/ajcn/nqz189 (PMC6821547; doi:10.1093/ajcn/nqz189)
Supplement: nqz189_Supplemental_File [file nqz189_supplemental_file.docx]

**Supplementary material to Louis-Auguste *et al.*:**

**Tryptophan, glutamine, leucine and micronutrient supplementation improve environmental enteropathy in Zambian adults: a randomized controlled trial**

**Supplementary methods**

1. *Assessment of lamina propria CD4+ T lymphocyte MTORC1 activity*

Endoscopic biopsies from the distal duodenum were immediately placed on ice in complete RPMI culture medium (Fisher Scientific, Loughborough, UK) at the time of endoscopy. Biopsies were agitated in 1mM EDTA for 15 minutes at 37°C to separate the epithelium which was discarded. The remaining lamina propria was macerated and lamina propria mononuclear cells were then enzymatically liberated using a solution containing 1 mg/mL collagenase D (Roche Diagnostics GmBH, Mannheim, Germany) and 20 μg/mL DNAse I (Roche Diagnostics GmBH, Mannheim, Germany) made up immediately before use in pre-warmed complete RPMI, and agitated for 40 minutes at 37 °C. The resulting cellular suspension was filtered through a 100 μm mesh, spun down and resuspended in either HBSS containing 5.5 mM glucose (the nutrient starved condition), or complete RPMI culture medium with supplemental L-glutamine (total Gln 4 mM; total glucose 11 mM; the nutrient rich condition). Both culture media also contained 50 μg/mL gentamicin and 100 iu/mL penicillin and streptomycin. Cells were incubated for 90 minutes before being fixed in 4 % paraformaldehyde followed by permeabilisation with 50 % methanol at –20 °C for at least 24 hours. Fixed & permeabilised cells were stained with fluorochrome-conjugated antibodies to phospho-4EBP1/PE (Cell Signalling Technology, Leiden, Netherlands), CD3/APC-Cy7 (clone SK7), CD4/PerCP-Cy5.5 (clone RPA-T4) & HLA-DR/APC (clone G46-6 or isotype control; all from BD Biosciences, Oxford, UK), and acquired on a FACSVerse cytometer (BD Biosciences, Oxford, UK).

1. *^1^H Nuclear magnetic resonance (NMR) spectroscopy-based metabolic phenotyping*

Once thawed, samples (400 μL) were combined with 200 μL of phosphate buffer (pH 7.4; 100% D_2_O) containing 1 mM of 3-trimethylsilyl-1-[2,2,3,3-^2^H_4_] propionate (TSP) as an internal standard and 2 mM sodium azide as a bacteriocide. Samples were mixed by vortexing and centrifuged at 10000 *g* for 10 minutes. The supernatant (550 μL) was transferred to a 5 mm internal diameter NMR tube. Samples were analysed by ^1^H nuclear magnetic resonance (NMR) spectroscopy using a 600 MHz Bruker NMR spectrometer operating at 300 K. For each urine sample a standard one-dimensional pulse sequence using the first increment of the NOE pulse sequence for water suppression was performed using 8 dummy scans followed by 128 scans collected into 64K data points. A mixing time of 10 ms was used with an acquisition time of 3.8 s and a recycle delay of 3.0 s. ^1^H NMR spectra were automatically corrected for phase and baseline distortions using Topspin 3.2 (Bruker Biospin). Chemical shifts in the spectra were referenced to the TSP peak at 0.0 ppm. Spectra were digitized using an in-house Matlab (version R2012a, The Mathworks, Inc.; Natwick, MA) script. Redundant spectral regions (those containing resonances derived from water and TSP) were removed and spectra were normalized to the total area.

**Supplementary table 1. Non-pathogenic enteric parasites in study participants.**

| Parasite | Number of stool samples |
| --- | --- |
| *Endolimax nana* | 28 |
| *Entamoeba coli* | 27 |
| *Blastocystis hominis* | 15 |
| *Iodamoeba bütschlii* | 7 |
| *Retortamonas intestinalis* | 5 |
| *Entamoeba hartmanni* | 1 |
| Total | **83** |
| No parasites identified (% of participants) | **58 (56.9)** |
| One or more parasites identified (% of participants) | **44 (43.1)** |

**Supplementary table 2. Multivariate linear regression model for change in villus height.**

|  | Univariate model | | Multivariate model | |
| --- | --- | --- | --- | --- |
|  | β | *P* | β | *P* |
| AA | 34.6 (1.0,68.1) | 0.04 | 38.7 (2.5,74.9) | 0.03 |
| MM | -10.9 (-45.8,24.0) | 0.5 |  |  |
| Body fat percent | -0.8 (-2.5,0.9) | 0.4 |  |  |
| Grip strength | 1.0 (-1.3,3.2) | 0.4 |  |  |
| MUAC | -1.3 (-6.1,3.4) | 0.6 |  |  |
| HIV status | -19.4 (-58.6,19.8) | 0.3 |  |  |

n=43. Abbreviations used: AA, amino acid supplementation; MM, multiple micronutrient supplementation; MUAC, mid-upper arm circumference.

**Supplementary table 3. Changes in markers of translocation and inflammation**

|  | Amino acid arm | | | Micronutrient arm | | |
| --- | --- | --- | --- | --- | --- | --- |
| Change in: | AA | Placebo | *P* | MM | Placebo | *P* |
| LPS (EU/ml) | -36 (-136, 86) | -24  (-91,58) | 0.64 | -55 (-165, 15) | 5 (-91, 86) | 0.11 |
| CRP (mg/l) | 0.05 (-0.8, 1.7) | -0.3 (-2.9, 2.0) | 0.26 | 0.06 (-0.8, 2.5) | -0.2 (-1.5, 0.8) | 0.19 |
| sCD14 (mg/l) | 0.3 (-0.07, 0.9) | 0.1 (-0.3, 0.7) | 0.28 | 0.1 (-0.3, 0.7) | 0.4 (-0.2, 0.8) | 0.37 |
| GLP2 | -0.21 (-0.63, 0.57) | -0.34 (-0.52, 0) | 0.44 | -0.25  (-0.55, 2.6) | -0.25  (-0.56, 0.58) | 0.66 |

Values are given as median (IQR) and *P* values shown were produced by the Wilcoxon rank sum test.

**Supplementary table 4. Significant urinary metabolites identified through metabonomic analysis: origins and putative roles.**

| Metabolite |  | Pathway | Origin | Effect on VH/MTORC1 in this study | Possible roles in enteropathy, stunting or nutrition | Ref |
| --- | --- | --- | --- | --- | --- | --- |
| β-hydroxy-β-methylbutyrate | HMB | Leu | Host | Positive correlation with VH and MTORC1 | Minor physiological leucine metabolite. Some evidence for promoting lean muscle mass. Positively correlated with VH in childhood EE. MTORC1 agonist. | 1,2 |
| Phenylacetyl-glutamine | PAG | Phe | Co-metabolite | Negative correlation with MTORC1 | Produced by hepatic conjugation of l-Gln with microbially derived phenylacetic acid. Correlated with VH in childhood EE. | 2,3 |
| Creatine |  |  | Host | Positive correlation with VH | Determined by muscle mass |  |
| Creatinine |  |  | Host | Positive correlation with VH | Determined by muscle mass |  |
| *N*-methylnicotinic acid | NMNA | B3 / Trp / IDO | Host |  | l-Trp / Niacin (B3) metabolite. Reduced levels observed in rat model of malnutrition. Correlated with childhood wasting and underweight. | 4,5 |
| 3-(3-hydroxy-phenyl)-3-hydroxy-propionic acid | HPHPA | Phe | Co-metabolite |  | Microbially derived l-Phe metabolite – particularly *Clostridia*. Possibly implicated in neuropsychiatric disorders. | 6,7 |
| β-amino-isobutyric acid | BAIBA | Val | Host |  | Lower levels associated with enhanced catch-up growth in childhood undernutrition. Anti-inflammatory and protective against oxidative stress and insulin resistance. Thermogenic. | 4 |
| *N*-methyl-2-pyridone-5-carboxamide | 2PY | Trp | Host | Positive correlation with VH and MTORC1 | Correlated with childhood stunting. | 4 |
| *N*-methyl-nicotinamide | NMND | B3 / Trp / IDO | Host |  | l-Trp / Niacin (B3) derivative. Higher levels associated with enhanced catch-up growth in childhood undernutrition. | 4 |
| 2-hydroxy-isobutyrate | 2HIB |  | Co-metabolite | Positive correlation with MTORC1 | Correlated with childhood stunting and VH in childhood EE. | 2,4 |
| Pantothenate |  | B5 | Host |  | Required for lipid biosynthesis. Correlated with childhood stunting. | 4 |
| Taurine |  | Cys | Co-metabolite | Positive correlation with VH | Low levels associated with childhood stunting. | 8 |
| 4-cresyl sulphate | 4CS | Tyr | Co-metabolite | Negative correlation with MTORC1 | Correlated with childhood stunting. | 4 |
| Citrate |  | TCA | Host |  | Negatively correlated with childhood stunting. | 4 |
| Succinate |  | TCA | Host |  | Correlated with VH in childhood EE. | 2 |
| Fumarate |  | TCA | Host |  | Unknown |  |
| Myo-inositol |  | PL | Host |  | Enhances insulin sensitivity. Precursor to membrane phospholipids. |  |
| Formate |  |  | Co-metabolite | Positive correlation with VH | Byproduct of acetate metabolism. |  |
| Hippurate |  |  | Co-metabolite | Negative correlation with MTORC1 | Correlated with childhood wasting and underweight. | 4 |
| 4-hydroxyhippurate |  |  | Co-metabolite |  | Correlated with VH in childhood EE. | 2 |
| Glycolate |  |  |  |  | Unknown |  |
| *Trans-*aconitate |  | TCA |  |  | Unknown |  |
| 3-indoxyl sulphate | 3IS | Trp | Co-metabolite |  | Correlated with childhood stunting and VH in childhood EE. | 2,4 |
| Choline |  |  |  |  | Required for lipid membrane stability and lipid metabolism. |  |
| Acetylcholine |  | TCA |  |  | Unknown |  |
| Guanidinoacetic acid | GAA |  |  | Positive correlation with VH | Creatine precursor; livestock muscle enhancer supplement | 9 |
| Dimethylamine |  |  | Co-metabolite | Positive correlation with MTORC1 |  |  |
| Dimethylglycine | DMG |  |  | Positive correlation with VH | Marker of choline flux. Negatively correlated with stunting. | 4 |

This table lists the significant metabolites identified through metabonomic analysis (see tables 5, 6 and 7 in main paper). Non-metabolite abbreviations: B3, niacin; B5, pantothenate; IDO, indolamine 2,3-dioxygenase; TCA, tricyclic acid cycle; PL, phospholipid synthesis.

**Supplementary table 5. Adverse events in AA vs placebo**

| Adverse event | Placebo (n) | AA (n) |
| --- | --- | --- |
| Abdominal fullness |  | 1 |
| Abdominal pain | 8 | 3 |
| Accidental ingestion of sachet |  | 1 |
| Anorexia |  | 2 |
| Backache | 5 | 4 |
| Body pains | 3 | 1 |
| Body weakness |  | 3 |
| Chest pain | 1 |  |
| Cough | 4 | 1 |
| Cough requiring oral antibiotics | 6 | 6 |
| Diarrhoea (severe)* | 5 | 2 |
| Diarrhoea (uncomplicated) | 5 | 8 |
| Dizziness | 5 | 2 |
| Dysmenorrhoea |  | 1 |
| Febrile illness requiring oral antibiotics | 1 | 1 |
| Feet swelling | 1 |  |
| Fever | 1 | 1 |
| Headache | 10 | 13 |
| Heart palpitations | 2 |  |
| Herpes zoster |  | 1 |
| Hunger | 6 | 15 |
| Incomplete abortion | 1 |  |
| Joint pains | 4 | 3 |
| Malaise | 1 |  |
| Malaria (self-diagnosed)** |  | 1 |
| Malarial illness** | 3 | 6 |
| Nausea | 5 | 10 |
| Odynophagia |  | 1 |
| Oversedation at endoscopy*** | 1 |  |
| Painful legs | 2 | 3 |
| Phlebitis at sedative injection site | 2 | 1 |
| Prolonged menstrual bleeding | 1 |  |
| Rash | 1 |  |
| Reduced mobility | 1 |  |
| Sleepy |  | 2 |
| Sneezing | 2 | 4 |
| Sore mouth | 1 |  |
| Sore throat | 1 |  |
| Sore throat requiring oral antibiotics | 1 | 1 |
| Toothache |  | 1 |
| Toothache requiring oral antibiotics | 1 |  |
| Borborygmi |  | 1 |
| Vomiting | 6 | 6 |
| Weakness | 1 | 1 |
| Weight loss |  | 1 |
| TOTAL | **98** | **108** |

*Diarrhoea classed as severe if required time off work / usual activities, lasted 3 or more days, was bloody, or required medical attention. None of the cases classed as severe were because of dysentery or requiring medical attention. ** There were no confirmed cases of malaria. ***Reversal agents not required.

**Supplementary table 6. Adverse events in MM versus placebo group**

| Adverse event | Placebo (n) | MM (n) |
| --- | --- | --- |
| Abdominal fullness | 1 |  |
| Abdominal pain | 4 | 7 |
| Accidental ingestion of sachet | 1 |  |
| Anorexia | 1 | 1 |
| Backache | 5 | 4 |
| Body pains | 2 | 2 |
| Body weakness |  | 3 |
| Chest pain |  | 1 |
| Cough | 2 | 3 |
| Cough requiring oral antibiotics | 4 | 8 |
| Diarrhoea (severe)* | 2 | 5 |
| Diarrhoea (uncomplicated) | 9 | 4 |
| Dizziness | 3 | 4 |
| Dysmenorrhoea |  | 1 |
| Febrile illness requiring oral antibiotics |  | 2 |
| Feet swelling | 1 |  |
| Fever | 1 | 1 |
| Headache | 8 | 15 |
| Heart palpitations | 1 | 1 |
| Herpes zoster |  | 1 |
| Hunger | 8 | 13 |
| Incomplete abortion | 1 |  |
| Joint pains | 4 | 3 |
| Malaise |  | 1 |
| Malaria (self-diagnosed)** | 1 |  |
| Malarial illness** | 5 | 4 |
| Nausea | 6 | 9 |
| Odynophagia | 1 |  |
| Oversedation at endoscopy*** | 1 |  |
| Painful legs | 3 | 2 |
| Phlebitis at sedative injection site | 1 | 2 |
| Prolonged menstrual bleeding | 1 |  |
| Rash |  | 1 |
| Reduced mobility |  | 1 |
| Sleepy |  | 2 |
| Sneezing | 2 | 4 |
| Sore mouth | 1 |  |
| Sore throat |  | 1 |
| Sore throat requiring oral antibiotics | 1 | 1 |
| Toothache | 1 |  |
| Toothache requiring oral antibiotics |  | 1 |
| Borborygmi | 1 |  |
| Vomiting | 5 | 7 |
| Weakness | 1 | 1 |
| Weight loss |  | 1 |
| TOTAL | **89** | **117** |

*Diarrhoea classed as severe if required time off work / usual activities, lasted 3 or more days, was bloody, or required medical attention. None of the cases classed as severe were because of dysentery or requiring medical attention. ** There were no confirmed cases of malaria. ***Reversal agents not required.

**Supplementary figure 1. Agreement between observers of small intestinal leak assessed by CLE.**


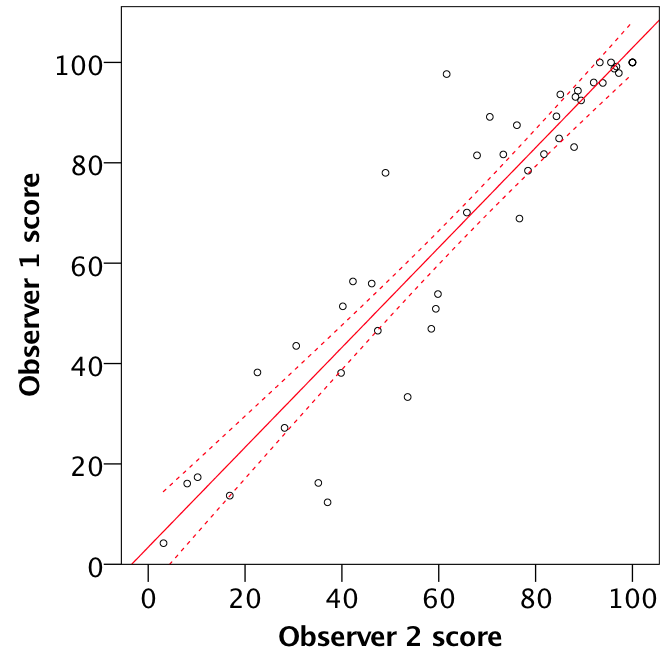


The proportion of fields demonstrating pathological endomicroscopic leak (Watson grade II or III; see methods in main paper) was assessed by two independent and blinded observers (PK and JLA). Spearman’s ρ = 0.94; *P* < 0.001.

**Supplementary figure 2. Compliance with AA and MM trial interventions**

Compliance was high: median (IQR) percentage of amino acid supplement packs consumed was 73 (60-86)% and of micronutrient capsules taken was 80 (67-88)%. The proportion of participants who consumed over 2/3 of all dispensed supplements was 74% in the micronutrient group, and 66% in the amino acid group. The correlation between MM and AA consumption was high (Spearman’s ρ 0.85, *P* < 0.001). Adjustment for the proportion of supplement consumed made no difference to the results and is not included in the data presented.

**Supplementary figure 3.** Correlation (Spearman’s ρ = 0.51; *P* = 0.001) between mTORC1 responsiveness in lamina propria CD4^+^ T lymphocytes and villus height (VH)

**Supplementary references**

1. Borack, M. S. & Volpi, E. Efficacy and Safety of Leucine Supplementation in the Elderly. *J. Nutr.* **146**, 2625S-2629S (2016).

2. Farràs, M. *et al.* Characterizing the metabolic phenotype of intestinal villus blunting in Zambian children with severe acute malnutrition and persistent diarrhea. *PLOS ONE* **13**, e0192092 (2018).

3. Aronov, P. A. *et al.* Colonic Contribution to Uremic Solutes. *J. Am. Soc. Nephrol.* **22**, 1769–1776 (2011).

4. Mayneris-Perxachs, J. *et al.* Urinary N-methylnicotinamide and β-aminoisobutyric acid predict catch-up growth in undernourished Brazilian children. *Sci. Rep.* **6**, (2016).

5. Wu, Z. *et al.* Urinary metabonomics study in a rat model in response to protein-energy malnutrition by using gas chromatography-mass spectrometry and liquid chromatography-mass spectrometry. *Mol. Biosyst.* **6**, 2157–2163 (2010).

6. Shaw, W. Increased urinary excretion of a 3-(3-hydroxyphenyl)-3-hydroxypropionic acid (HPHPA), an abnormal phenylalanine metabolite of Clostridia spp. in the gastrointestinal tract, in urine samples from patients with autism and schizophrenia. *Nutr. Neurosci.* **13**, 135–143 (2010).

7. Xiong, X., Liu, D., Wang, Y., Zeng, T. & Peng, Y. Urinary 3-(3-Hydroxyphenyl)-3-hydroxypropionic Acid, 3-Hydroxyphenylacetic Acid, and 3-Hydroxyhippuric Acid Are Elevated in Children with Autism Spectrum Disorders. *BioMed Res. Int.* **2016**, (2016).

8. Semba, R. D. *et al.* Child Stunting is Associated with Low Circulating Essential Amino Acids. *EBioMedicine* **6**, 246–252 (2016).

9. Ostojic, S. M. Advanced physiological roles of guanidinoacetic acid. *Eur. J. Nutr.* **54**, 1211–1215 (2015).
